# Sepsis Burden in a Major Romanian Emergency Center—An 18-Year Retrospective Analysis of Mortality and Risk Factors

**DOI:** 10.3390/medicina61050864

**Published:** 2025-05-08

**Authors:** Florentina Mușat, Dan Nicolae Păduraru, Alexandra Bolocan, Cosmin-Alexandru Palcău, Andrei-Alexandru Bunea, Daniel Ion, Octavian Andronic

**Affiliations:** 1 Carol Davila University of Medicine and Pharmacy, Faculty of Medicine, General Surgery Department, University Emergency Hospital of Bucharest, 050098 Bucharest, Romania; florentina.musat@drd.umfcd.ro (F.M.); bolocan.alexa@gmail.com (A.B.); alexandru-cosmin.palcau@drd.umfcd.ro (C.-A.P.); dr.daniel.ion@gmail.com (D.I.); octavian.andronic@umfcd.ro (O.A.); 2 Department of Computer Science, National University of Science and Technology Politehnica Bucharest, 060042 Bucharest, Romania; andrei.bunea@stud.acs.upb.ro; 3 Innovation and eHealth Center, Carol Davila University of Medicine and Pharmacy, 010451 Bucharest, Romania

**Keywords:** sepsis, epidemiology, risk factors, comorbidities, mortality, outcomes

## Abstract

*Background and Objectives:* Sepsis is a leading cause of morbidity and mortality worldwide, yet data from Central and Eastern Europe remain scarce. Our study aims to address the scarcity of information regarding the characteristics and mortality rates of patients with sepsis by reporting recent data from one of the largest emergency centers in Romania over an 18-year period (2007–2024). *Materials and Methods:* A retrospective analysis was conducted on 12,089 adult patients diagnosed with sepsis at the University Emergency Hospital of Bucharest. Patients were identified using International Classification of Diseases (ICD-10) codes and free-text diagnosis. Demographic and clinical data were extracted, including comorbidities, interventions, and mortality outcomes. Associations between comorbidities and in-hospital mortality were assessed using odds ratios (ORs) and 95% confidence intervals (CIs). *Results:* The study population had a mean age of 68.7 years, with a slight predominance of males (50.9%). In-hospital mortality was 53.9%, and 30-day mortality reached 85.1%. The most common comorbidities were diabetes (27.2%), chronic kidney disease (14.0%), and cancer (12.9%). Pneumonia (OR = 2.08, 95% CI: 1.89–2.28), cirrhosis (OR = 1.69, 95% CI: 1.40–2.03), and chronic obstructive pulmonary disease (OR = 1.50, 95% CI: 1.27–1.77) were strong predictors of mortality, while diabetes was associated with a slightly lower risk (OR = 0.90, 95% CI: 0.83–0.97). *Conclusions:* Sepsis-related mortality in Romania is higher than reported in Western Europe and North America, resembling trends in resource-limited settings. Targeted early recognition, antimicrobial stewardship, and improved intensive care units (ICU) resource allocation are crucial for reducing mortality. Multicenter studies and microbiological analyses are needed to further understand sepsis outcomes in this region.

## 1. Introduction

Sepsis represents a complex syndrome manifested by organ dysfunction as a result of a dysregulated response to an infection. This life-threatening condition claims millions of lives each year globally, despite advancements in diagnosis and medical care, and was declared an important worldwide health concern by the World Health Organization in 2017 [1]. Sepsis incurs a substantial humanistic and economic burden, with 48.9 million cases and over 11.0 million associated deaths globally in 2017 according to a Global Burden of Disease study [2]. In 2017, the age-standardized incidence rate (ASIR) of sepsis was reported to be highest in low- and middle-income countries from Africa (particularly sub-Saharan Africa at 1527.2 cases per 100,000 individuals), Oceania, and South Asia (1117.9 and 982.9 cases per 100,000 individuals, respectively) and lowest in high-income countries from North-America and Western Europe (247.8 and 167.8 cases per 100,000 individuals, respectively) [2]. In Romania, according to the same study, the sepsis ASIR was reported at 270.0 cases per 100,000 individuals.

The Intensive Care over Nations Audit (ICON) study [3] reported substantial differences in sepsis prevalence among continents, with rates ranging from 13.6% in South Asia to 39.3% in East and Southeast Asia. The prevalence of sepsis in the ICON study was 30.5% in Eastern Europe and 31.3% in Western Europe. These values suggest that sepsis remains a significant health challenge in European ICUs, including those in Romania.

Sepsis is associated with 20% of total deaths worldwide [2]. The global age-standardized mortality rate (ASMR) among patients with sepsis was 148.1 per 100,000 individuals in 2017 and showed a deep discrepancy between high-income countries (30.3 deaths per 100,000 individuals) and low- and middle-income countries (470.2, 451.4, and 289.6 deaths per 100,000 individuals in Sub-Saharan Africa, Oceania, and South Asia, respectively) [2]. By comparison, the ASMR among patients with sepsis in Romania was 54.1 per 100,000 individuals [2]. According to a meta-analysis of studies conducted in North America, Europe, and Australia between 2009 and 2019, the 30-day mortality rate in patients with sepsis was 24.4% (95% confidence interval 21.5–27.2%) and was slightly higher in Europe (23.6%) compared to North America (19.6%) and Australia (18.7%) [4]. However, the mortality rates reported in some recent studies can vary widely, as shown in a recent review, which reported a range between 11.5% and 59.1% in cohorts of patients with sepsis used in training machine learning models for predicting survival outcomes [5].

Analyses of epidemiology, patient characteristics, risk factors, diagnosis, and management techniques are critical to understand areas of unmet need, encourage further research, and influence health policies and clinical decisions in sepsis. Studies reporting sepsis-related incidence and mortality rates from Central and Eastern European countries are scarce. Moreover, the epidemiological data summarized so far should be interpreted with caution due to several limitations such as lack of robust country-level data, imprecision of administrative coding and diagnosis reporting, and high heterogeneity among methods and reporting among different studies [2,4,6]. Our study aims to address the scarcity of information regarding characteristics and mortality rates of patients with sepsis by reporting recent data from one of the largest emergency centers in Romania.

## 2. Methods

Patient records were extracted from the hospital information system (HIS) of the University Emergency Hospital of Bucharest in collaboration with specialists from the HIS provider—InfoWorld. The Bucharest Emergency University Hospital is the largest emergency hospital in Romania, with 1099 beds and more than 49,000 patients treated in 2024 [7]. The Info World hospital management platform for managing patient records was introduced in healthcare units throughout Romania in 2000 and implemented at the Bucharest Emergency University Hospital in 2000.

Adult patients with sepsis, admitted to the University Emergency Hospital in Bucharest between 1 January 2007 and 31 December 2024 were identified based on a pre-specified list of International Classification of disease (ICD-10) codes (see Supplementary Table S1). Patients who had a primary or secondary sepsis diagnosis ICD code or had a sepsis diagnosis listed among the conditions recorded as free-text were selected. In our study, the term ”free-text diagnosis” refers to clinical diagnoses entered manually by physicians into non-structured text fields within the hospital’s electronic medical record (EMR) system, rather than selecting from standardized ICD-10 diagnostic codes. These entries typically describe the patient’s condition in the clinician’s own words at the time of discharge or during hospitalization (e.g., “urosepsis”, “septic shock secondary to pneumonia”), often providing additional clinical nuance that may not have been fully captured by codified diagnoses.

All data were fully anonymized to preclude any possibility of direct patient identification. Each patient was assigned a unique, randomly generated identifier to facilitate subsequent statistical analyses while preserving anonymity. Patients with one-day hospitalization or admitted to the neonatology department or under 18 years old were excluded. During the COVID-19 pandemic, the hospital included in our study remained designated as a non-COVID emergency and trauma center and was not converted into a COVID-19 referral hospital. As such, the majority of patients with primary severe acute respiratory syndrome coronavirus 2 (SARS-CoV-2) infection were managed in other dedicated facilities.

However, we did include patients in our cohort who were incidentally diagnosed with COVID-19 as a secondary pathology, but COVID-19 was not the primary cause of sepsis in any of the included cases. Therefore, while the pandemic period is covered within our study timeline, its direct impact on sepsis incidence or mortality in this specific patient population was limited. Patients with SARS-CoV-2 infection were identified based on the ICD code U07.1, which was mandatorily included in the diagnostic records of all individuals confirmed to carry the virus. This coding practice was consistently applied throughout the pandemic period, ensuring that all COVID-19-positive patients were systematically recorded and accurately included in the dataset for analysis.

**Figure 1:**
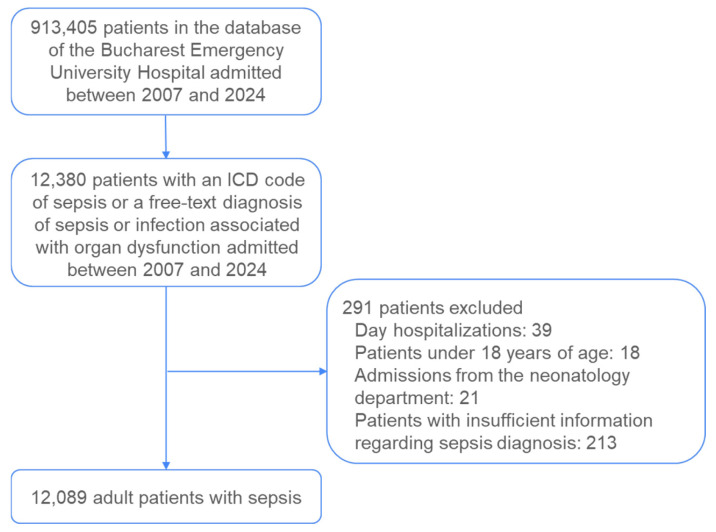
Patient inclusion flowchart.

A total of 12,380 patient records were extracted from the hospital’s information system for the period spanning 2007 to 2024. These records were subsequently analyzed according to a predefined set of inclusion and exclusion criteria (Figure 1).

Day hospitalizations (*n* = 39), patients under 18 years of age (*n* = 18), admissions from the neonatology department (*n* = 21), and patients lacking a sepsis diagnosis (*n* = 213) were excluded. The presence of sepsis was verified across three diagnostic categories: the primary diagnosis code, the secondary diagnosis code, and the free-text diagnosis. Patients who did not have sepsis indicated in any of these three diagnostic fields but who had a concurrent diagnosis of infection combined with organ dysfunction and subsequently died were classified as septic and included in the final dataset. Ultimately, 12,089 adult patients with sepsis were included in the analysis.

Information, including demographic characteristics, clinical characteristics, primary and secondary diagnostic, laboratory results, interventions, hospitalization length, and status at discharge, was extracted in a tabulated format. Cases were classified as surgical or non-surgical. The types of variables extracted are detailed in Table 1.

An independent samples *t*-test was conducted to compare the average age between women and men. A chi-square test of independence (χ^2^) was used to examine whether there was a statistically significant association between in-hospital death and sex, age, presence of surgical intervention, and presence of comorbidities. The odds ratio (OR) was used to quantify the strength and direction of association between comorbidities and mortality. To identify the factors independently associated with mortality following sepsis cases, we applied a multivariate logistic regression model. We used the following covariates: sex, age, presence of a surgical intervention, and the presence of each of the nine types of comorbidities (based on diagnostic codes recorded during the hospitalization period). The selection of covariates (age, sex, surgical status, and major comorbidities) was based on a priori clinical relevance and evidence from previous sepsis studies demonstrating their strong and consistent association with mortality risk. These variables were reliably recorded across the full 18-year study period, allowing for robust and unbiased inclusion in the model.

To maintain confidentiality and prevent patient identification, all collected data were fully anonymized prior to analysis. Additionally, secure data storage systems were employed, incorporating encryption protocols to safeguard against unauthorized access and potential security breaches. Compliance with data protection regulations, including adherence to national and international legal frameworks such as the General Data Protection Regulation, was strictly upheld throughout the study. The investigations were carried out following the rules of the Declaration of Helsinki of 1975, which was revised in 2013 [8]. The study received approval from the Ethics Committee of the Carol Davila University of Medicine and Pharmacy (Approval code: 6098/21.03.2025.) and from The University Emergency Hospital of Bucharest—approval code: 36964/17.06.2022.

## 3. Results

Over the 18-year period, there were 913,405 total hospital admissions, with 12,089 septic patients, resulting in an overall sepsis prevalence of 1.32% (Table 2). Sepsis prevalence has shown a steady and substantial increase over time, from 0.26% in 2007 to 2.82% in 2024. From 2007 to 2012, the prevalence increased gradually from 0.26% to 0.70%. Between 2013 and 2019, sepsis prevalence continued to rise, reaching 1.87% by 2019. A sharp increase was observed in 2017 (4.65%), possibly due to changes in documentation or classification. Despite a drop in total hospitalizations during the COVID-19 pandemic (e.g., 28,069 in 2020 vs. 49,048 in 2019), the number of septic patients remained high, resulting in elevated sepsis prevalence—2.71% in 2020 and 2.82% in 2021 and 2024. The most recent years show a stable but elevated prevalence between 2.54% and 2.86%

The study population consists of 12,089 septic patients, with a slight predominance of men (6157, 50.9%) over women (5932, 49.1%). The average age of the cohort is 68.7 years (standard deviation [SD] 16.0). Women were statistically significantly older (71.5 years) compared to men (66.0 years, *p* < 0.001). Patient age ranges from a minimum of 18 years to a maximum of 102 years, with a median age of 71 years (Table 3).

Of the cohort, 24.6% (*n* = 2685) received mechanical ventilation from 2011 to 2024, while 6.6% (*n* = 725) underwent renal replacement therapy. The patient group was categorized into surgical cases (27.5%, 3320 patients) and non-surgical cases (72.5%, 8769 patients), reflecting a predominance of non-surgical presentations in this septic cohort (Table 3).

Among the study population, diabetes was the most prevalent comorbidity, affecting 27.2% of patients (*n* = 3291), followed by cardiovascular and renal conditions, with atherosclerosis reported in 14.1% (*n* = 1707) and chronic kidney disease in 14.0% (*n* = 1686). Cancer was present in 12.9% (*n* = 1558) of patients, with non-hematological malignancies (9.0%, *n* = 1092) being more frequent than hematological cancers (3.9%, *n* = 466). Other notable comorbidities included chronic obstructive pulmonary disease (COPD) in 5.3% (*n* = 641) and cirrhosis in 4.3% (*n* = 519). In terms of infectious complications, Clostridium difficile infection was identified in 5.2% (*n* = 626).

Additionally, conditions commonly associated with sepsis were reported at high rates, including pneumonia in 20.7% (*n* = 2503) and peritonitis in 7.3% (*n* = 883).

**Figure 2:**
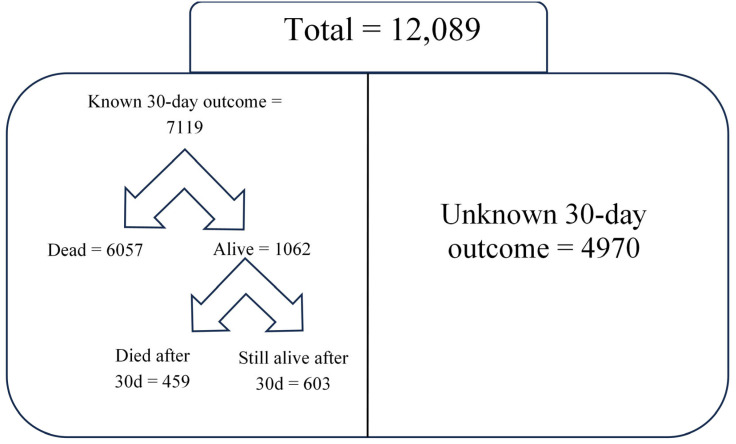
The left side represents the 7119 patients whose 30-day outcome was known—this includes those who died within 30 days or were still hospitalized at 30 days (459 who died after 30 days and 603 still alive after 30 days). The right side represents the 4970 patients who were discharged alive before 30 days but for whom no follow-up data were available—thus their survival status at 30 days is unknown. These two groups are mutually exclusive and together make up the total study population of 12,089 patients.

The overall in-hospital mortality in the study population was substantial, with a total of 6516 deaths (53.9%), including 3397 deaths among women (57.3%) and 3119 among men (50.7%). The mortality rate among women was significantly higher compared to that among men (chi-square test, χ^2^ = 52.82, *p* < 0.001); however, when adjusting for sex, receipt of surgery, and comorbidities, men were at higher odds for death compared to women (adjusted odds ratio (OR) 1.13; 95% confidence interval (CI) 1.04–1.22). Among the 6516 overall in-hospital deaths, the majority (6057/6516) occurred within 30 days of hospitalization. Within the entire cohort of patients with sepsis, 603 were alive 30 days after hospitalization; 4970 patients were discharged alive within 30 days of admission, and we have no post-discharge follow-up information available to verify whether they remained alive at 30 days. To avoid underestimating or overestimating 30-day mortality, we only calculated this outcome in the subgroup of patients for whom we could reliably determine the 30-day vital status. This subgroup included 6057 patients who died within 30 days of admission, 459 who died after 30 days (confirmed alive at day 30), and 603 still alive after 30 days (confirmed alive at day 30). Thus, the 30-day mortality rate among patients who either died or were still alive and hospitalized at 30 days (7119 patients with known 30-day status) was 85.1% (Table 3, Figure 2).

**Figure 3:**
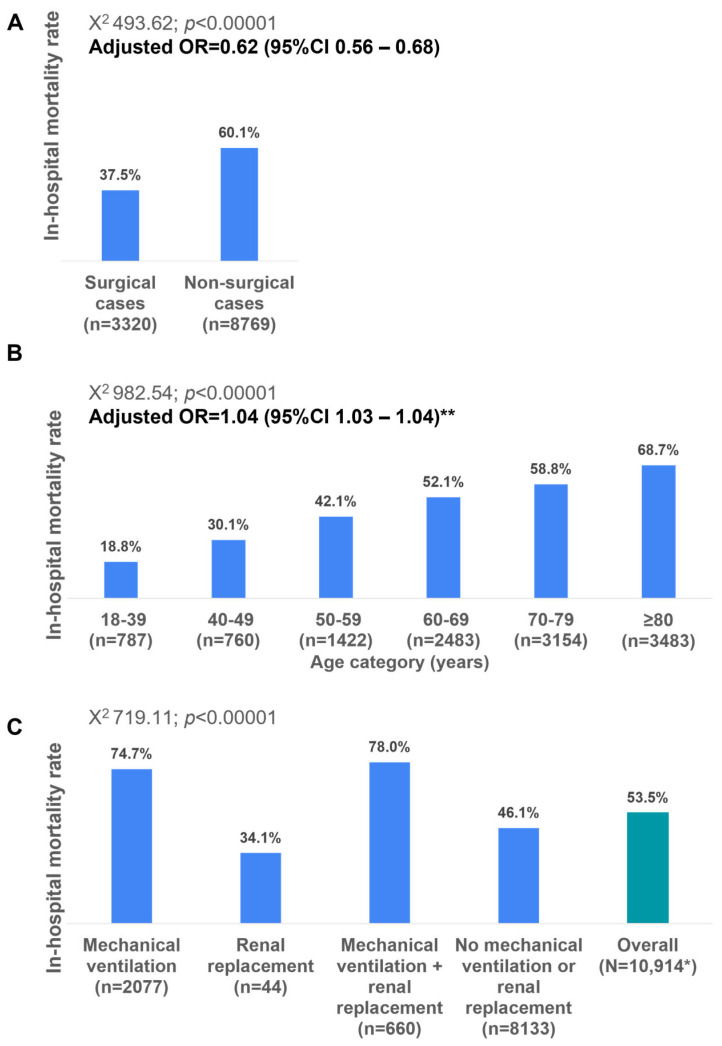
In-hospital mortality rates based on surgical/non-surgical categories (panel (**A**)), age categories (panel (**B**)), and receipt of mechanical ventilation with or without renal replacement (panel (**C**)). * Among patients recorded between 2011 and 2024. ** Variables included in the adjustment: age, sex, surgical status, presence of comorbidities. Age is considered as a continuous variable in the multivariate analysis. OR—odds ratio; CI—confidence interval.

The in-hospital mortality rate was significantly lower among the surgical cases compared to the non-surgical cases (37.5% versus 60.1%; adjusted OR 0.62; 95%CI 0.56–0.68) and among older age categories (Figure 3A,B). The in-hospital mortality rates vary significantly across different age categories, demonstrating a clear increasing trend with advancing age. Youngest age group (18–39 years) had the lowest mortality rate at 18.8% (*n* = 787). Mortality gradually increases with age, surpassing 50% in patients aged 60 years and older; the highest value (66.7%) is observed in patients aged ≥80 years (*n* = 3483) (Figure 3B).

In-hospital mortality varied significantly based on the need for intensive care unit (ICU) interventions. Patients requiring both mechanical ventilation and renal replacement therapy had the highest mortality rate (78.0%), followed by those requiring mechanical ventilation alone (74.7%). In contrast, the mortality was significantly lower in patients requiring only renal replacement therapy (34.1%) and those who did not require either intervention (48.1%) (Figure 3C).

**Figure 4:**
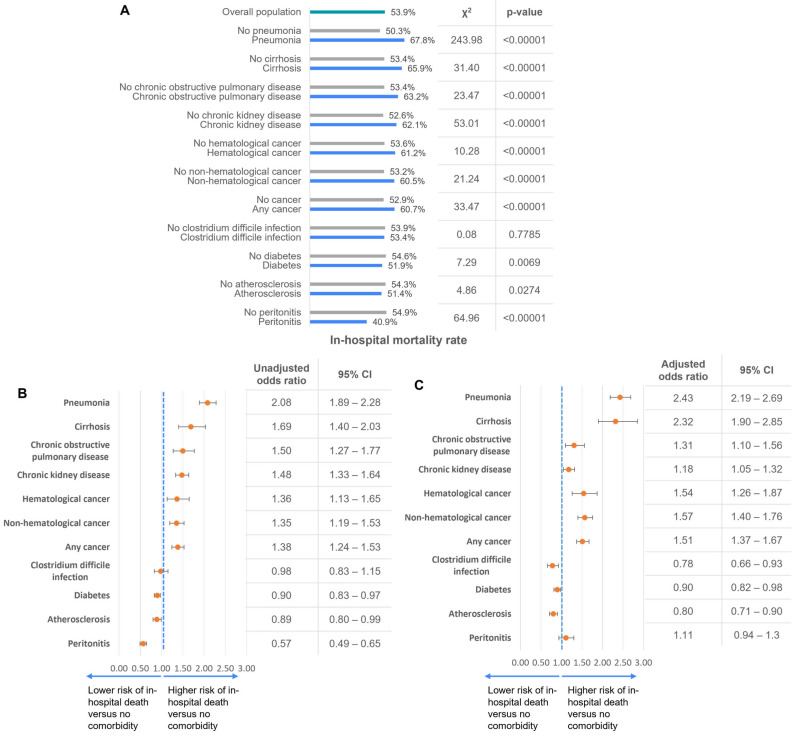
In-hospital mortality rates among patients with sepsis and various comorbidities. (**A**) Bar chart showing stratified in-hospital mortality rates across the study population (n = total cohort), comparing patients with and without specific conditions. Chi-square statistics and *p*-values indicate the strength and significance of observed differences. (**B**) Unadjusted odds ratios (ORs) for in-hospital mortality by clinical and comorbid characteristics. Forest plot presenting univariate logistic regression results. Confidence intervals (95% CI) are provided for each factor. (**C**) Adjusted odds ratios (ORs) for in-hospital mortality after multivariable logistic regression. Forest plot displaying adjusted ORs with 95% CIs for each variable after controlling for confounders.

The in-hospital mortality was significantly associated with the presence of comorbidities. The in-hospital mortality rate was significantly higher among those with cancer (60.7%), cirrhosis (65.95%), chronic kidney disease (62.1%), chronic obstructive pulmonary disease (63.2%), and pneumonia (67.8%) compared to those without these comorbidities. Patients with diabetes (51.9%), clostridium difficile infection (53.4%), and atherosclerosis (51.4%) had similar in-hospital mortality rates compared to those without these comorbidities, while in patients with peritonitis, in-hospital mortality was lower (40.9%) compared to those without peritonitis (Figure 4A–C).

After adjusting for age, sex, receipt of surgery, and comorbidities in multivariate analysis, pneumonia and cirrhosis were the strongest predictors, indicating a more than twofold increased mortality risk (adjusted OR = 2.43, 95% CI: 2.19–2.69 and adjusted OR = 2.32, 95% CI: 1.90–2.85, respectively). Chronic kidney disease (adjusted OR = 1.18, 95% CI: 1.05–1.32) and chronic obstructive pulmonary disease (COPD) (adjusted OR = 1.31, 95% CI: 1.10–1.56) were also significantly associated with increased mortality risk. Additionally, any cancer (adjusted OR = 1.51, 95% CI: 1.37–1.67), hematological cancer (adjusted OR = 1.54, 95% CI: 1.26–1.687), and non-hematological cancer (adjusted OR = 1.57, 95% CI: 1.40–1.76) were associated with higher risk (Figure 4C).

Conversely, the impact of diabetes, atherosclerosis, and clostridium difficile infection on in-hospital mortality was marginal in both the unadjusted and adjusted analyses. Notably, peritonitis was associated with a reduced risk of in-hospital death in the unadjusted analysis; however, after adjusting for age, sex, receipt of surgery, and presence of other comorbid conditions, the association was no longer significant (Figure 4B,C). These findings highlight the substantial impact of respiratory, hepatic, renal, and oncological conditions on patient outcomes, emphasizing the need for targeted interventions in high-risk groups.

Table 4 presents data on COVID-19 cases and related mortality recorded in the study hospital from 2020 to 2024. During this period, 417 patients with confirmed COVID-19 were identified, of whom 251 died, resulting in an overall in-hospital mortality rate of 60.2% among COVID-positive patients. The overall prevalence of COVID-19 among patients admitted during the pandemic period (2020–2024, *n* = 5196) was 8.03%.

The highest number of COVID-19 cases occurred in 2022 (*n* = 135), followed by 2021 (n = 108) and 2020 (*n* = 93). The highest mortality rates were observed in 2021 (65.7%) and 2022 (63.7%), likely reflecting the impact of more severe COVID-19 waves and limited treatment options during those years. In contrast, the number of cases and mortality rates declined in 2023 (54.2%) and 2024 (57.6%), likely due to improved patient management, greater vaccine coverage, and the emergence of less virulent viral variants.

Although the hospital was not a designated COVID-19 center, it remained an emergency and trauma hospital throughout the pandemic and admitted patients who developed COVID-19 as a secondary diagnosis but not as the primary cause of sepsis.

**Figure 5:**
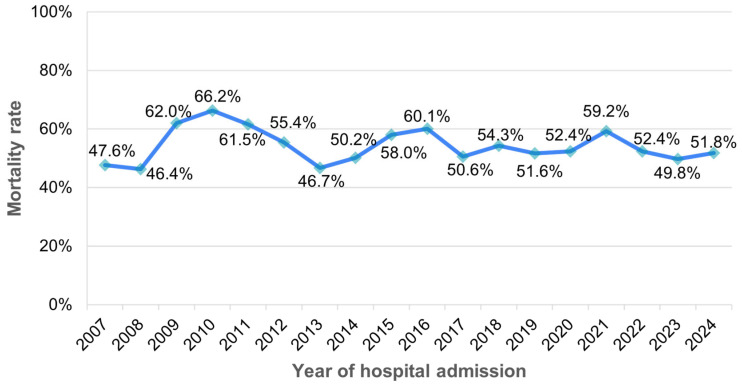
In-hospital mortality rates among patients with sepsis between 2007 and 2024.

Between 2007 and 2024, the yearly in-hospital mortality rates among patients with sepsis varied between 46.4% (in 2008) and 66.2% (2010), with no obvious trends over time (Figure 5).

**Figure 6:**
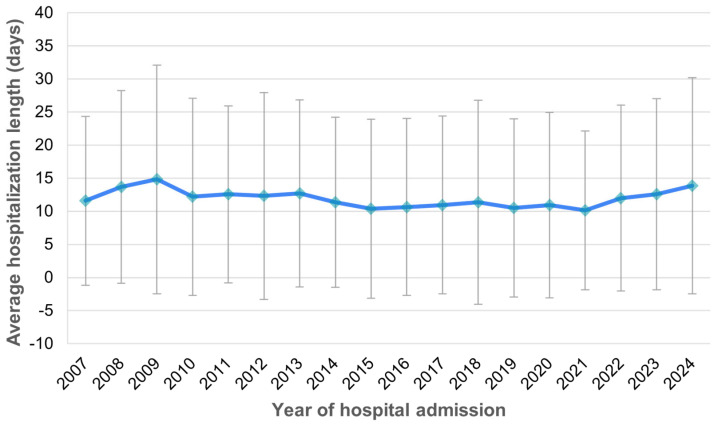
Hospitalization length among patients with sepsis between 2007 and 2024.

The mean duration of hospitalization was 11.8 days (SD 14.3 days), ranging from a minimum of 0 to a maximum of 213 days, with a median length of stay of 8 days. The average hospitalization length remained consistent over time between 2007 and 2024 (Figure 6).

Table 5 presents microbiological data from 2410 patients who were tested for infectious agents, revealing the distribution of pathogens identified among this subgroup. The most frequently detected organisms were Clostridium difficile (25.98%), Staphylococcus spp. (19.83%), *Escherichia coli* (17.01%), and *Klebsiella* spp. (16.51%). Other commonly identified pathogens included Gram-negative bacteria (11.99%), *Streptococcus* spp. (5.89%), *Pseudomonas* spp. (7.47%), and *Candida* spp. (4.27%). Less prevalent organisms included *Proteus* spp. (3.32%), anaerobes (3.65%), *Acinetobacter* spp. (1.54%), and fungi (1.91%), with *Salmonella* (0.29%), *Mycoplasma* (0.58%), and *Haemophilus influenzae* (0.33%) being rare.

## 4. Discussion

The present study highlights the burden of sepsis among patients admitted to a large emergency center in Romania. From 2007 to 2012, the prevalence increased gradually from 0.26% to 0.70%. This period likely reflects limited awareness, evolving diagnostic criteria, and lower detection or reporting rates for sepsis. Sepsis prevalence continued to rise, reaching 2.82% by 2024. The increase may be attributed to improved recognition of sepsis, changes in coding practices, and a growing hospital focus on infection-related complications. The data reveal a marked increase in sepsis prevalence over time, suggesting improved detection, diagnosis, and documentation practices, but also possibly reflecting growing sepsis burden in an aging and increasingly comorbid patient population. Our findings align with the key observations reported by Yasser Sakr et al. [3] in their study on sepsis in ICU patients worldwide. Both studies confirm the significant variability in mortality rates and the influence of underlying comorbidities on patient outcomes. However, the differences across geographies in sepsis outcomes and risk factors may be due to disparities in healthcare infrastructure, patient demographics, and clinical practice.

The data presented here were collected between the years 2007 and 2024, a period during which the definition of sepsis underwent two major revisions. Initially, sepsis was defined based on the systemic inflammatory response syndrome (SIRS) criteria (Sepsis-1) [9], which was later refined in 2001 (Sepsis-2) [10] to emphasize organ dysfunction as a defining characteristic of severe sepsis. In 2016, a paradigm shift occurred with the introduction of the Sepsis-3 definition [11], which abandoned the SIRS-based approach and instead defined sepsis as life-threatening organ dysfunction due to a dysregulated host response to infection. This change also introduced the quick Sequential Organ Failure Assessment (qSOFA) score as a tool for early risk stratification. Given these evolving definitions, earlier cases in the dataset may have been identified and categorized differently than more recent cases, affecting comparisons across time periods. Moreover, shifts in diagnostic criteria and clinical recognition may have influenced the reported incidence, severity stratification, and treatment approaches. These factors must be acknowledged when interpreting the trends and outcomes in the present study, as they may contribute to variations in sepsis-related mortality and other clinical parameters.

The noticeable increase in sepsis prevalence in 2017 coincided with several institutional and systemic changes at the Emergency University Hospital of Bucharest. It is important to consider the publication of the Sepsis-3 definition in 2016. Sepsis-3 simplified the diagnostic criteria for sepsis, focusing on life-threatening organ dysfunction due to infection and abandoning the more complex SIRS-based Sepsis-1/Sepsis-2 models. Sepsis-3 also introduced the qSOFA score, a simple bedside tool usable outside of the ICU to rapidly identify patients at risk of sepsis. Although Sepsis-3 was not immediately and uniformly adopted across all institutions, a lag period of approximately one year is plausible before clinical practice adapted. We believe that, by 2017, this simplified framework may have facilitated greater recognition and classification of septic patients in our hospital, further contributing to the observed spike in sepsis prevalence. Additionally, during this period, national policy changes led Romania’s Ministry of Health to promote stricter ICD-10 coding for sepsis, prompting our hospital to train clinicians and coders accordingly. Starting in 2017, the Emergency University Hospital of Bucharest launched internal campaigns to improve sepsis recognition and documentation, especially in the Emergency and Internal Medicine Departments and the ICU, likely increasing formal diagnosis and coding.

Our study population consisted primarily of older adults, with an average age of 69 years. Similarly, other studies [3,12,13] found that sepsis patients were generally older.

Patients with sepsis required significant healthcare resources, as a quarter of the population received mechanical ventilation and the average hospitalization duration was 12 days. The ICON study reported that 68% of septic patients required mechanical ventilation, while 9.2% underwent renal replacement therapy (RRT) [3]. Our study showed lower rates of ICU interventions, with 24.6% of patients receiving mechanical ventilation and 6.6% requiring RRT. This suggests that the severity of cases in our study might differ from the global ICU population analyzed in ICON or that the ICU admission criteria and treatment practices differ between Romania and other regions.

Our findings show that sepsis-related mortality was higher among women than men, contrasting with the Global Burden of Disease study [2], which reported a higher age-standardized mortality rate (ASMR) in males (164.2 vs. 134.1 per 100,000 in 2017), and the ICON study [3], which found no significant sex-based differences in sepsis outcomes. This discrepancy may be explained by the older age of female patients in our cohort, as advanced age was independently associated with increased mortality in both our study and previous research [13]. Further investigation is required to determine whether this pattern is unique to our study population or indicative of broader epidemiological trends.

Our study reported an in-hospital mortality rate of 53.9%, significantly higher than the overall hospital mortality of 35.3% reported by ICON [3]. The ICON study also noted significant geographical differences in mortality rates. Oceania had the lowest ICU mortality (11.9%) and hospital mortality (19.3%). Africa had the highest ICU mortality (39.5%) and hospital mortality (47.2%). Western Europe and Eastern Europe had hospital mortality rates of 33.3% and 44.8%, respectively. North America had lower mortality rates (25.2% hospital mortality), likely reflecting better access to advanced ICU care and sepsis treatment protocols. The disparity in mortality rates may be attributed to several factors, including differences in patient selection criteria, the severity of illness at presentation, variations in clinical management, or healthcare resource constraints in our setting. Additionally, our study reported a 30-day mortality of 85.1%, which is significantly higher than the figures seen in ICON. This discrepancy could be due to differences in case severity, inclusion criteria, or the length of follow-up in our study compared to ICON. Country-level factors such as gross national income (GNI), ICU bed availability, healthcare resources, and access to advanced sepsis treatments have been shown to significantly influence sepsis outcomes, with studies highlighting the impact of delayed ICU admission, inadequate staffing, and resource limitations on patient survival [3,12]. Hospital-wide antimicrobial stewardship programs (ASPs) were not fully implemented during the majority of the study period. Empiric antibiotic choices were often unguided by antibiograms, and delays or inappropriate initial therapies may have contributed to adverse outcomes. This reflects broader national challenges in antimicrobial resistance control and stewardship in Romanian hospitals. Romania has among the lowest ICU bed densities in Europe. In our hospital, ICU admission is prioritized for surgical and trauma patients, often limiting timely access for septic patients with rapidly evolving organ dysfunction. Many such patients are treated in general wards with limited capacity for intensive monitoring or escalation of care. Low nurse-to-patient ratios and limited sepsis-specific training across non-ICU settings reduce the ability to monitor for deterioration or respond rapidly, both of which are critical in sepsis care. The elevated mortality in our study underscores the need for targeted interventions to improve early sepsis recognition and management strategies in Romania. It also suggests that sepsis outcomes in Romania may be influenced by challenges similar to those faced by low- and middle-income countries, such as limited ICU resources, delays in sepsis recognition, and differences in antimicrobial resistance patterns.

Our study and the ICON study also share common findings regarding risk factors associated with mortality [3]. Both studies identified age, the presence of chronic illnesses such as cancer and cirrhosis, and the need for mechanical ventilation or renal replacement therapy as strong predictors of death among septic patients. However, while the ICON study highlighted the independent role of Acinetobacter spp. infection as a significant risk factor for mortality, we did not specifically analyze the impact of microbiological profiles on patient outcomes. Given the regional variations in pathogen distribution and antimicrobial resistance patterns reported by Sakr et al. [3], future studies within our setting should explore the influence of specific infectious agents on sepsis outcomes.

A recent systematic literature review examined the influence of type 2 diabetes on sepsis outcomes and found conflicting evidence regarding its impact on mortality [14]. While poor glycemic control is a known contributor to severe infections, certain antidiabetic treatments, including insulin, metformin, and thiazolidinediones, have been linked to a reduced incidence and mortality in sepsis. Some studies suggest that chronic hyperglycemia may induce immune adaptation, potentially lowering mortality rates in diabetic patients with sepsis [14]. However, despite this possible protective effect, an increased risk of acute kidney injury in diabetic septic patients is well-documented [14].

The association between diabetes and sepsis-related mortality remains uncertain, as studies have reported mixed results. While some large observational and retrospective cohort studies have found higher mortality rates in diabetic patients with infections and sepsis [15], others have found no significant association [16,17,18], and one study even suggested improved survival in diabetic patients [19].

Our study supports these findings, as diabetes had a marginal impact on in-hospital mortality (OR = 0.90, 95% CI: 0.83–0.97). This suggests that diabetes might not be a direct risk factor for increased mortality in sepsis, potentially due to better patient management, the protective effects of antidiabetic medications, or other confounding factors. However, due to the retrospective nature of our study and the limitations of the available dataset, we did not have access to detailed clinical information such as glycemic control levels, treatment regimens, or the presence of diabetes-related complications. Moreover, given the large sample size of 12,089 patients, incorporating such granular stratification would require extensive clinical documentation that was not consistently recorded across the 18-year period. While we acknowledge that a sensitivity analysis based on these variables would provide valuable insight, it was beyond the scope of the current analysis. Given the complexity of the relationship between diabetes and sepsis outcomes, further research is needed to clarify the mechanisms behind this association and to determine whether specific diabetes treatments contribute to improved survival in septic patients.

Epidemiological data indicate that bacterial infections of the lungs (68% of sepsis cases), followed by intra-abdominal infections (22% of cases), represent the leading causes of sepsis [20,21,22]. Furthermore, evidence from previous research suggests that the site of infection may independently influence patient survival in sepsis, as some studies have reported that sepsis associated with pneumonia is associated with higher mortality rates among critically ill patients [23]. Although this hospital was not a designated COVID-19 center, COVID-19 still affected a notable number of patients, many of whom were admitted for other emergencies. The high mortality rate (60.2%) highlights the vulnerability of this population, possibly due to underlying conditions, delayed presentation, or coexisting pathologies. These figures confirm that, while not the primary focus of sepsis in this cohort, COVID-19 had a measurable impact on outcomes and should be acknowledged when interpreting mortality trends during the pandemic years. On the other hand, in the study by Li Q (2021) [22], patients in the abdominal sepsis group exhibited a higher ICU mortality rate compared to those in the pulmonary sepsis group. However, their length of stay (LOS) in the ICU was significantly shorter (both *p* < 0.001). Despite this difference, no statistically significant variation was observed between the two groups regarding overall hospital mortality or total hospital LOS (both *p* > 0.005).

In our study, patients with peritonitis had a lower in-hospital mortality rate (40.9%) compared to those without peritonitis (54.9%). Conversely, patients with pneumonia exhibited a higher in-hospital mortality rate (67.8%) compared to those without pneumonia (50.3%).

In our retrospective study of 2410 culture-positive patients, Gram-negative organisms predominated, with Escherichia coli (17.0%), Klebsiella spp. (16.5%), and Pseudomonas spp. (7.5%) being most common. This is similar to the findings from a large, prospective study [3], where Gram-negative pathogens accounted for 67.1% of isolates. Gram-positive organisms, particularly Staphylococcus spp. (19.8%) and Streptococcus spp. (5.9%), were also frequently identified in both studies. Notably, Clostridium difficile was detected in 26.0% of our patients—a prevalence substantially higher than that reported in the ICON study, where it was not specifically listed, suggesting differences in diagnostic focus or epidemiologic context. Fungal infections were less common in our cohort (6.2% overall compared to 12.9% in the ICON data). These differences likely reflect variations in study design, microbiological practices, and regional epidemiology. While global and Western European studies on sepsis prevalence and mortality are well-documented, there are limited data from Central and Eastern Europe. This study provides one of the largest datasets on sepsis in Romania, filling a gap in regional epidemiological research.

Prior research suggests that sepsis mortality varies widely by region, with lower rates in high-income countries. This study highlights significantly higher in-hospital and 30-day mortalities (53.9% and 85.1%, respectively) than those reported in Western Europe and North America, offering insights into the impact of healthcare system limitations on sepsis outcomes.

One of the major strengths of this study is the large sample size, which included 12,089 patients. The inclusion of such a substantial number of cases enhances the statistical power and reliability of the findings, allowing for more robust conclusions regarding sepsis epidemiology, patient characteristics, and outcomes. This study spans 18 years (2007–2024), providing long-term data on sepsis incidence, mortality, and hospital length of stay, which is rarely documented in previous Romanian or regional studies.

Another advantage of the study is that the identification of patients with sepsis relied not only on ICD coding but also on free-text documented diagnosis. Also, when sepsis was not explicitly mentioned as a diagnosis, cases of infection associated with organ dysfunction were included, thus ensuring comprehensive identification of all relevant cases.

A limitation of this study is that it was conducted in a single-center setting, which may limit the generalizability of the findings to other hospitals, regions, or healthcare systems. The patient population, clinical practices, availability of resources, and institutional protocols at this center may not fully reflect those of other ICUs, particularly in different geographical areas, hospital types, or healthcare infrastructures. As a result, the study’s findings may be influenced by site-specific factors, such as variations in sepsis management, antimicrobial stewardship, and ICU admission criteria. This limitation highlights the need for multicenter studies to better capture the broader variability in sepsis epidemiology and treatment outcomes across diverse healthcare settings.

The definition of sepsis and its classification under the International Classification of Diseases (ICD) system have changed over time, leading to inconsistencies in the data. However, we did not retrospectively adjust or reclassify cases according to evolving definitions (Sepsis-1, Sepsis-2, or Sepsis-3). Our approach was to identify sepsis cases based on how they were originally documented in the hospital information system, using a combination of ICD diagnostic codes and free-text diagnosis entries. This method was chosen to reflect real-world clinical practice and preserve the consistency of case identification as it occurred over time. While we recognize that this may introduce some heterogeneity in the classification of sepsis, any attempt to retrospectively standardize the diagnoses according to newer criteria would have required access to detailed clinical and organ dysfunction data, which were not available for all patients. The accuracy of coding can also vary significantly, potentially misclassifying or underreporting sepsis cases.

The study used mortality records rather than patient-level clinical data. Unlike hospital-based studies, there was no way to verify organ failure criteria directly from clinical records. Also, due to the retrospective design and the nature of the hospital’s electronic records, we did not have consistent access to variables such as median time to ICU admission or standardized severity scores (e.g., SOFA, Acute Physiology and Chronic Health Evaluation—APACHE II) across the entire cohort. These data were either not systematically recorded or were unavailable for a large portion of the 18-year study period. The absence of time-to-intervention and severity metrics restricts our ability to evaluate their influence on survival. In addition, more than half of our patients were discharged within 30 days of hospitalization and had no available follow-up information after discharge.

Another limitation of this study is that we were unable to provide detailed results on microbiological profiles and antimicrobial resistance patterns due to the lack of data on the total number of patients who underwent pathogen testing. While we did include data on the identified pathogens and associated mortality rates for cases with available microbiological results (as shown in the attached tables), this does not reflect the true prevalence of specific microorganisms in our cohort, as we do not have information on the total number of patients who were microbiologically tested. Additionally, some patients had multiple pathogens isolated, further complicating prevalence estimation. Given this limitation, the microbiological data presented must be interpreted with caution and cannot be generalized to the entire study population. This information gap may lead to an incomplete understanding of sepsis etiology and hinder efforts to optimize empirical antibiotic strategies.

The 25.98% prevalence of Clostridium difficile among the 2410 microbiologically tested patients appears elevated compared to typical sepsis cohorts. This finding likely reflects selection bias in the subset of patients who underwent microbiological testing rather than a true prevalence across the entire septic population. Specifically, C. difficile testing is typically reserved for patients with gastrointestinal symptoms (e.g., persistent diarrhea, ileus) or suspected healthcare-associated colitis, both of which are common complications in elderly or immunocompromised patients with sepsis. The high proportion of C. difficile in this tested subgroup may therefore reflect targeted diagnostic practices rather than generalized testing. Additionally, it is important to note that this subgroup of 2410 patients does not represent a random sample but a clinician-selected group based on suspected or atypical infectious presentations. This could have led to the overrepresentation of C. difficile, as it is a recognized cause of both primary and secondary sepsis, particularly in patients with recent antibiotic exposure or prolonged hospitalization.

Future studies should ensure systematic microbiological testing and standardized reporting of resistance patterns to enhance the accuracy and clinical applicability of the findings.

## 5. Conclusions

This study presents a comprehensive 18-year analysis of sepsis in a large Romanian emergency hospital, revealing an alarmingly high in-hospital mortality rate. Key predictors of mortality included pneumonia, cirrhosis, chronic kidney disease, and chronic obstructive pulmonary disease, while diabetes was associated with a slightly lower risk. Compared to international data, sepsis mortality in Romania remains significantly higher, aligning more closely with the trends seen in resource-limited healthcare settings. These findings underscore the urgent need for enhanced early recognition and management strategies to improve outcomes for sepsis patients.

## Figures and Tables

**Table 1:** Variables extracted from the hospital database.

Category of Variables	Details
Demographic and administrative data	◦ Identification Code: a unique, randomly generated code assigned to each patient to ensure anonymity and facilitate data tracking ◦ Age and Sex ◦ Admission Date and Discharge Date ◦ Discharge Status: dead or alive
Diagnostic and procedural information	◦ Diagnoses recorded both as free text and as standardized diagnostic codes ◦ Codes corresponding to any mechanical ventilation and renal replacement procedures

**Table 2:** The annual number of hospitalized patients, the number of septic patients, and the calculated sepsis prevalence (%) from 2007 to 2024 in a large Romanian emergency hospital.

Year	Total No. of Hospitalized Patients	Total No. of Septic Patients	Sepsis Prevalence (%)
**2007**	57,332	147	0.26
**2008**	64,328	274	0.43
**2009**	64,270	363	0.56
**2010**	62,452	391	0.63
**2011**	63,592	364	0.57
**2012**	61,543	428	0.7
**2013**	59,863	514	0.86
**2014**	52,801	500	0.95
**2015**	48,829	685	1.4
**2016**	46,649	729	1.56
**2017**	45,893	755	4.65
**2018**	47,408	825	1.74
**2019**	49,048	918	1.87
**2020**	28,069	760	2.71
**2021**	30,896	871	2.82
**2022**	37,892	1085	2.86
**2023**	45,539	1155	2.54
**2024**	47,001	1325	2.82
**Total**	913,405	12,089	1.32

**Table 3:** Patient characteristics. SD—standard deviation; IQR—interquartile range.

Dataset Characteristics	N = 12,089
Mean age (SD)	68.7 (16.0) Women: 71.5 (15.7) Men: 66.0 (18.8)
Median age (range, IQR)	71 (18–102, 60–81) Females: 75 (18–102, 64–83) Males: 68 (18–100, 56–78)
Women, *n* (%)	5932 (49.1%)
Men, *n* (%)	6157 (50.9%)
Sepsis as main diagnosis, *n* (%)	4561 (37.7%)
Sepsis as secondary diagnosis, *n* (%)	7085 (58.6%)
Sepsis as free-text diagnosis or infection associated with organ dysfunction, *n* (%)	443 (3.6%)
Only mechanical ventilation, *n* (%, 2011–2024, N = 10,914) ***	2077 (19%)
Only renal replacement therapy, *n* (%, 2011–2024, N = 10,914) ***	44 (0,4%)
Mechanical ventilation and renal replacement, *n* (%, 2011–2024, N = 10,914) ***	660 (6%)
No mechanical ventilation or renal replacement, *n* (%, 2011–2024, N = 10,914) ***	8133 (74,5%)
Surgical cases, *n* (%)	3320 (27.5%)
Non-surgical cases, *n* (%)	8769 (72.5%)
Any cancer, *n* (%)	1558 (12.9%)
Hematological cancer, *n* (%)	466 (3.9%)
Non-hematological cancer, *n* (%)	1092 (9.0%)
Diabetes, *n* (%)	3291 (27.2%)
Cirrhosis, *n* (%)	519 (4.3%)
Chronic kidney disease, *n* (%)	1686 (14.0%)
Chronic obstructive pulmonary disease, *n* (%)	641 (5.3%)
Clostridium difficile infection, *n* (%)	626 (5.2%)
Atherosclerosis, *n* (%)	1707 (14.1%)
Peritonitis, *n* (%)	883 (7.3%)
Pneumonia, *n* (%)	2503 (20.7%)
In-hospital mortality, *n* (%)	6516 (53.9%)
30-day mortality, *n* (%, N = 7119) *	6057 (85.1%)
30-day mortality, men (%, N = 3452)	2888 (83.66%)
30-day mortality, women (%, N = 3667)	3169 (86.41%)
Mean hospitalization, days (SD, N = 12,089) **	11.8 (14.3)
Median hospitalization, days (range, N = 12,089) **	8 (0–213)

* Among patients with a follow-up of at least 30 days. ** Among inpatients. *** Data regarding ventilator use and renal replacement were recorded as codified procedures in the Info World system only after the year 2011.

**Table 4:** COVID-19 cases and related mortality recorded in the study hospital from 2020 to 2024.

	2020	2021	2022	2023	2024	Total
Total no. of COVID-19 cases	93	108	135	48	33	417
No. of deceased COVID-19 cases	49	71	86	26	19	251
Mortality of COVID-19-positive patients	52.7%	65.7%	63.7%	54.2%	57.6%	60.2%

**Table 5:** Microbiological data from a subgroup of 2410 culture-positive-infected patients.

		Pathogen Prevalence Among Tested Patients
Total no of tested patients	2410	
*Salmonella* spp.	7	0.29
*Streptococcus* spp.	142	5.89
*Staphylococcus* spp.	478	19.83
Gram negative	289	11.99
*Escherichia coli*	410	17.01
*Pseudomonas* spp.	180	7.47
*Klebsiella* spp.	398	16.51
*Proteus* spp.	80	3.32
*Mycoplasma pneumoniae*	14	0.58
*Haemophilus influenzae*	8	0.33
*Acinetobacter* spp.	37	1.54
*Anaerobes*	88	3.65
*Candida* spp.	103	4.27
Fungi	46	1.91
*Clostridium difficile*	626	25.98

## Data Availability

The datasets presented in this article are not readily available because the data are part of an ongoing study.

## Supplementary Materials

The following supporting information can be downloaded at: https://www.mdpi.com/article/10.3390/medicina61050864/s1, Table S1: The pre-specified list of International Classification of Disease (ICD-10) codes used to extract patients diagnosed with sepsis from the University Emergency Hospital of Bucharest Information System (InfoWorld). Total number of patients and the number of deceased patients that received at least on of this codes as a primary or secondary diagnosis.
