# Supplementary material for: Sepsis Burden in a Major Romanian Emergency Center—An 18-Year Retrospective Analysis of Mortality and Risk Factors
Source: Medicina (Kaunas). 2025 May 8;61(5):864. doi: 10.3390/medicina61050864 (PMC12113612; doi:10.3390/medicina61050864)
Supplement: Supplementary file 1 [file medicina-61-00864-s001.zip › medicina-3622700-supplementary.pdf]

Supplementary Table S1. The pre-specified list of International Classification of Disease (ICD-10) codes used to extract patients diagnosed with sepsis from the University Emergency Hospital of Bucharest Information System (InfoWorld). Total number of patients and the number of deceased patients that received at least on of this codes as a primary or secondary diagnosis.

		Total	Deceased
A02.1	Sepsis due to Salmonella	5	1
A26.7	Sepsis due to Erysipelothrix	0	0
A32.7	Listerial sepsis	0	0
A40.0	Sepsis due to Group A Streptococcus	9	1
A40.1	Sepsis due to Group B Streptococcus	9	3
A40.2	Sepsis due to Group D Streptococcus	7	1
A40.8	Other types of sepsis due to Streptococci	50	23
A40.9	Unspecified streptococcal sepsis	23	8
A41.0	Sepsis due to Staphylococcus aureus	207	41
A41.1	Sepsis due to other specified staphylococci	102	23
A41.2	Sepsis due to other unspecified staphylococci	34	15
A41.3	Sepsis due to Haemophilus influenzae	6	2
A41.4	Sepsis due to anaerobes	78	18
A41.50	Sepsis due to unspecified Gram-negative bacteria	76	20
A41.51	Sepsis due to Escherichia coli	256	34
A41.52	Sepsis due to Pseudomonas	75	26
A41.58	Sepsis due to other Gram-negative organisms	150	51
A41.8	Other specified types of sepsis	7420	4629
A41.9	Unspecified sepsis	3401	1504
A42.7	Actinomycotic sepsis	4	2
B37.7	Sepsis due to Candida	30	15
T81.42	Procedure-related sepsis	18	3
O88.3	Obstetric pyemic and septic embolism	3	2
	TOTAL	11963	6422
